# MatCol: a tool to measure fluorescence signal colocalisation in biological systems

**DOI:** 10.1038/s41598-017-08786-1

**Published:** 2017-08-21

**Authors:** Matloob Khushi, Christine E. Napier, Christine M. Smyth, Roger R. Reddel, Jonathan W. Arthur

**Affiliations:** 1 Bioinformatics Unit, Children’s Medical Research Institute, The University of Sydney, Westmead, NSW Australia; 2 Cancer Research Unit, Children’s Medical Research Institute, The University of Sydney, Westmead, NSW Australia; 3 Gene Therapy Unit, Children’s Medical Research Institute, The University of Sydney, Westmead, NSW Australia

## Abstract

Protein colocalisation is often studied using pixel intensity-based coefficients such as Pearson, Manders, Li or Costes. However, these methods cannot be used to study object-based colocalisations in biological systems. Therefore, a novel method is required to automatically identify regions of fluorescent signal in two channels, identify the co-located parts of these regions, and calculate the statistical significance of the colocalisation. We have developed MatCol to address these needs. MatCol can be used to visualise protein and/or DNA colocalisations and fine tune user-defined parameters for the colocalisation analysis, including the application of median or Wiener filtering to improve the signal to noise ratio. Command-line execution allows batch processing of multiple images. Users can also calculate the statistical significance of the observed object colocalisations compared to overlap by random chance using Student’s t-test. We validated MatCol in a biological setting. The colocalisations of telomeric DNA and TRF2 protein or TRF2 and PML proteins in >350 nuclei derived from three different cell lines revealed a highly significant correlation between manual and MatCol identification of colocalisations (linear regression R^2^ = 0.81, P < 0.0001). MatCol has the ability to replace manual colocalisation counting, and the potential to be applied to a wide range of biological areas.

## Introduction

Protein-DNA and protein-protein interactions are known to be markers and regulators of cellular and biological processes. For instance, the colocalisation of telomeric DNA and promyelocytic leukaemia (PML) protein is a marker of cells that utilise the alternative lengthening of telomeres (ALT) mechanism to maintain telomere length^1^, while the E3 ubiquitin ligase Mdm2 binds and negatively regulates the tumour suppressor p53^2^. Fluorescence microscopy can be used to visualise protein, DNA, and cell structures labelled with different fluorophores. The overlap of signal between the different channels can be analysed in the resultant images, and this serves as a measure for colocalisation of the biological entities labelled by the fluorophores.

Two types of quantitative colocalisation measures are currently used. The first type of measure is pixel intensity-based coefficients where overlapping fluorescent pixel intensities of two channels are used to calculate Pearson, Manders (M1 or M2), Li or Costes coefficients^3–5^. A number of commercial tools are available for studying these coefficients, as well as various open source ImageJ plugins, the most cited of which is JACoP^6, 7^. However, a researcher may experience one or more limitations as discussed here. The pixel intensity-based coefficients provide significantly varied results depending on the method selected, background pixel pre-processing, and whether a region of interest (ROI)-based approach is taken into account^8, 9^. Coefficients can be reported within individual ROIs, therefore, it is important to select biologically relevant ROIs. However, the inability to automatically select ROIs makes analysis of a large number of images extremely difficult^10^. Background can vary significantly in different portions of the images; therefore, global thresholding to remove background is not a suitable option as discussed by Regeling *et al*., as well as Adler and Parmryd^11, 12^. Different coefficients report colocalisation using a range of 0 to 1 or −1 to 1, which may lead to investigators considering different coefficients significant^8, 13^. For example, 0.51 may indicate a strong colocalisation to one researcher and a moderate colocalisation to another researcher. Finally, the tools used in pixel intensity-based coefficients do not report the exact number of colocalised objects found per ROI.

The second type of measure is an object-based counting of colocalised objects. In this method, if a segmented object on one channel having a specified number of overlapping pixels with a segmented object on the other channel then this is counted as a colocalisation^7, 14, 15^. The two types of colocalisation methods should not be considered competing as both could be used to address different quantification needs. Some results have demonstrated the usefulness and statistical robustness of such object-based quantification of molecules in cell biology^7^. Thus, object-based quantification would be more appropriate for studying colocalisation of small objects such as ALT-associated PML bodies (APBs), defined as the colocalisation of PML protein and telomeric DNA, as presented in this study. There is, however, a lack of tools available that can report the exact count of segmented objects in one channel that are colocalised with those in another channel. The ImageJ macro developed by Moser *et al*. calculates object-corrected Pearson coefficient^9^. In this method, objects are segmented with a threshold algorithm and Pearson coefficient weighted by the fraction of colocalisation. However, the macro does not report the count of colocalised objects. Verdoodt *et al*. devised a Fiji-based macro that can count colocalised objects, however, the link to download the code is broken^16^. CellProfiler^17^ provides another object-based colocalisation counting method, but the thresholding techniques available to analyse colocalisations did not work well for our images. Determining the optimal tool for image processing is a challenge for researchers in the biological sciences.

To address the limitations of currently available colocalisation tools, we have developed a novel object-based colocalisation tool called MatCol. The improvements made include automatically selecting ROIs, removing background by a novel thresholding algorithm, and calculating the statistical significance of the colocalisations.

## Results

### General MatCol features

**Figure 1:**
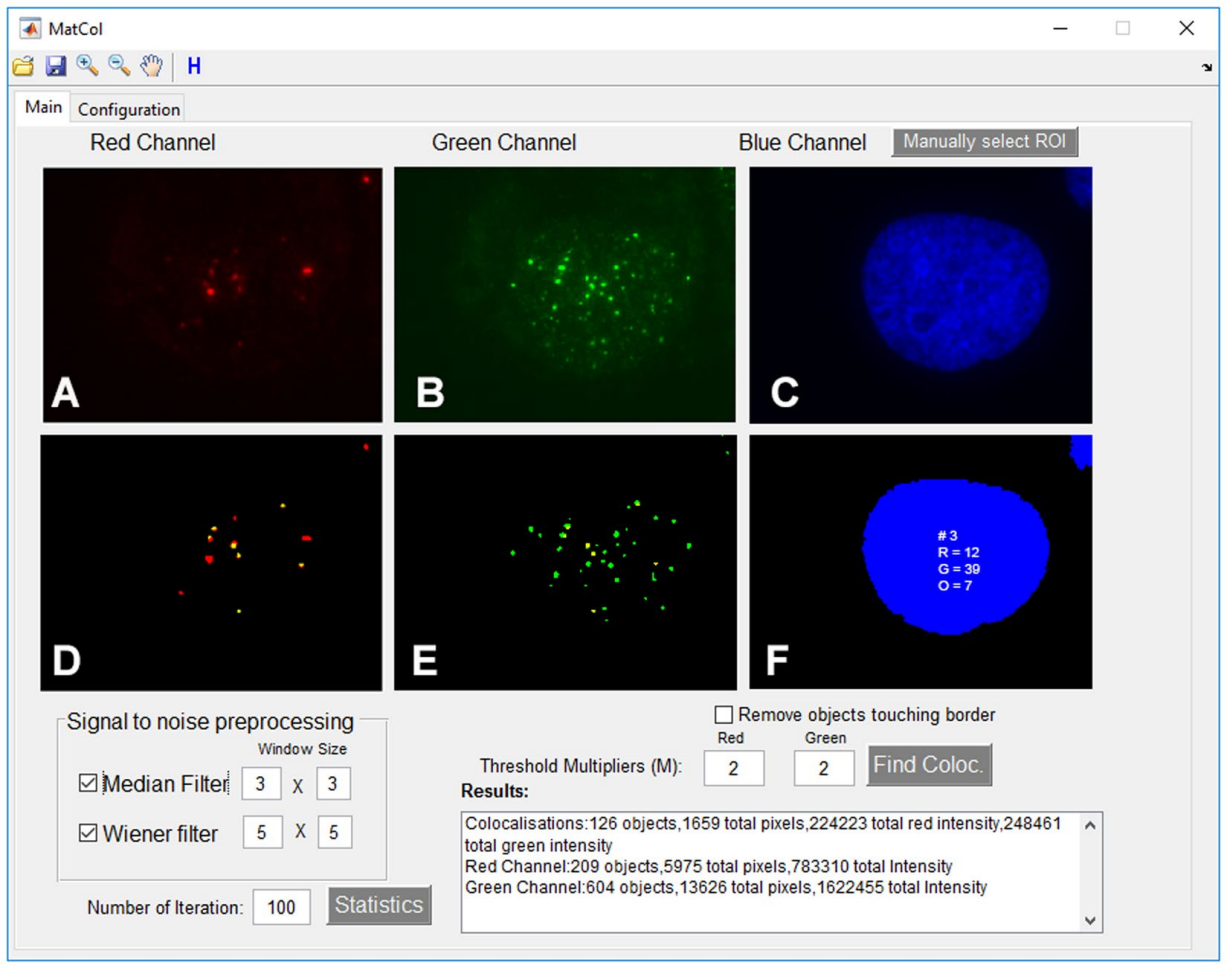
The MatCol graphical user interface (GUI). Six windows showing the red (**A**), green (**B**), and blue (**C**) channels in a single nucleus, along with their respective binary versions after removing the background (**D**,**E**,**F**). The screen shot has been zoomed to better display colocalised objects in the ROIs. Colocalisations of the two channels are shown in yellow in windows D and E. The coded ROI binary mask (**F**) enumerates the ROI sequence number (#), the red (“R”) and green (“G”) signals, as well as their overlap (“O”) in this single nucleus. The Results panel describes the features of the entire image, which has numerous ROIs. The remaining MatCol features in the GUI are described in the Results section. The entire image from which **A–F** was taken is shown in Supplementary Figure S1.

MatCol has a graphical user interface (GUI) that helps visualise an area of interest and fine tunes various parameters, if required, in order to complete an analysis of the defined area (Fig. 1). When an RGB TIFF image is opened in MatCol, the image is split across the red, green, and blue channels (Fig. 1A–C). MatCol is designed to study object-based colocalisation instead of pixel intensity-based correlation. In order to correctly recognise the object of interest, de-noising options (median and Wiener filters, and threshold multipliers) are provided in the GUI. Threshold multiplier is a user defined constant which is designed to filter the background as explained in the Equation 1 in the Methods. Removal of background reduces the identification of false-positive signals and also helps in correctly identifying the volume and edges of the object of interest images^18^. Default optimal threshold multiplier and window sizes for median and Wiener adaptive filters are those that worked well in various test images with different level of noise, and users are able to adjust the de-noising options as required. Generally, the criteria for determining protein locations become more stringent as the threshold multiplier increases. Pressing the “Find Coloc.” button results in each ROI image being labelled with red or green where the signal is above the threshold level (Fig. 1D,E). Overlapping red and green signals are coloured yellow in each ROI. Under the Configuration tab, the user can set the percentage or minimum number of pixels that must spatially overlap between the two channels in order to report a colocalisation of two objects (Supplementary Figure S2). The ROI binary mask (Fig. 1F) shows the ROI sequence number, and the total number of red, green and overlapping signals. Automatic selection of ROIs is the default setting; however, ROIs can be selected manually by clicking the “Manually select ROI” button. MatCol automatically selects ROI from the blue channel. If the ROI needed to be selected from red or green channel then images can be pre-processed using ImageJ/Fiji, as described in the Supplementary Information S1. In addition, objects touching the border are automatically included. These border-touching regions can be excluded by checking the “Remove objects touching border” checkbox and running the analysis again.

The “Save” button in the toolbar saves all channels in separate lossless TIFF files at the location of the originally opened file, appending the channel name to the filename to avoid overwriting the original data. Zooming and panning on any window is synchronised to all windows allowing easy comparison of the applied filters.

Subsequent to running the analysis, the Results window shows information regarding the colocalisations, and the red and green channels: the total number of objects, the total number of pixels, and the localised pixel intensity sum as described in the Methods section.

### Statistical significance of colocalisation

The statistical significance of the observed object colocalisations against overlap by random chance is computed using a one sample Student’s t-test^19, 20^. The randomisation process was achieved using Monte Carlo simulation^21^. After identifying the number of colocalised objects, use of the “Statistics” button randomly scatters the detected objects, keeping their size and shape consistent in their respective red or green channel within the ROIs. MatCol then counts the colocalised objects resulting from this random distribution of the objects. The random scattering is repeated according to the number of iterations specified by the user and the significance of colocalisation is calculated, as described in the Methods section.

### ROI-based background intensity selection

**Figure 2:**
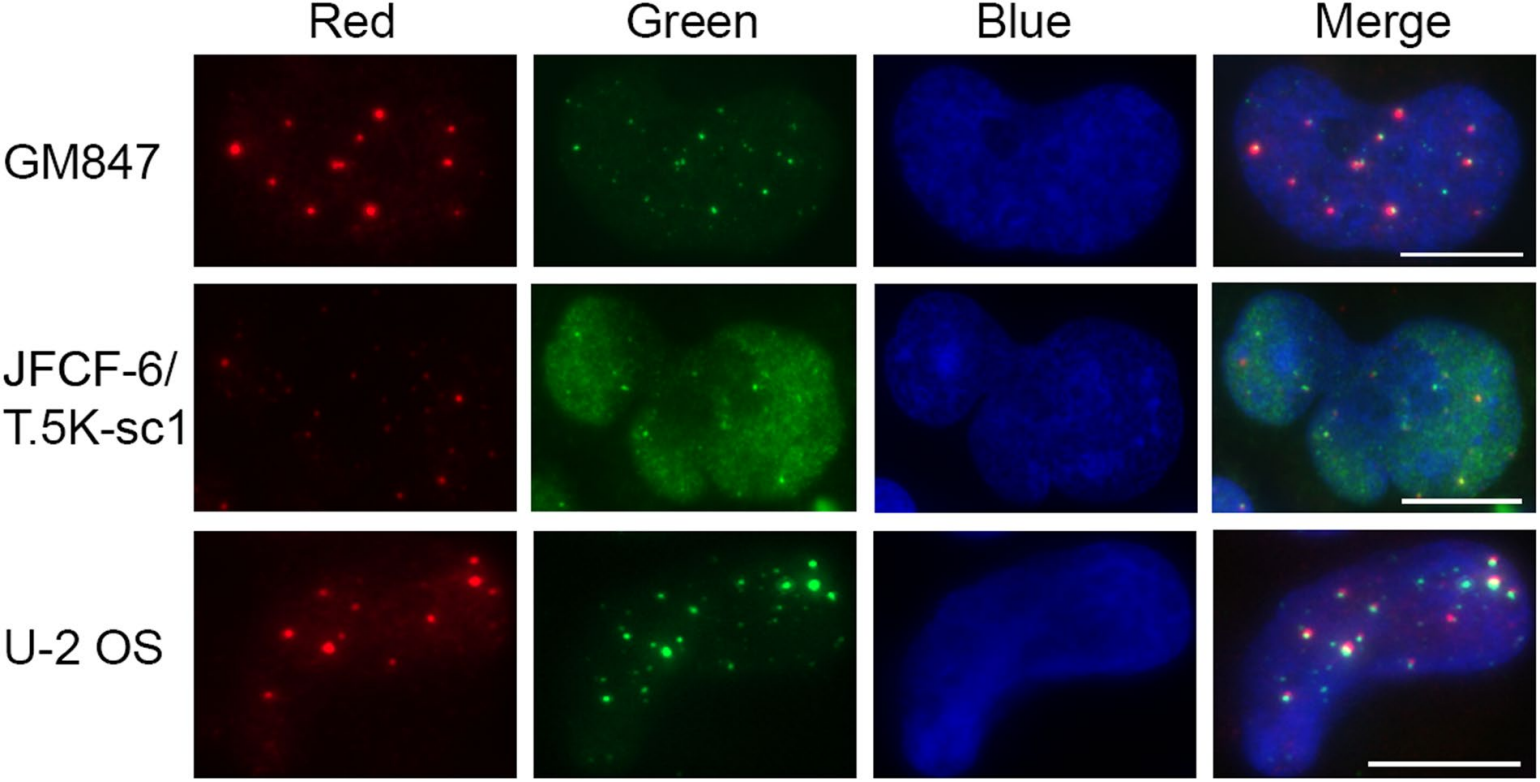
Background varies among cell lines and channels. Examples of three nuclei with varying degrees of background in the red and green channels. Cell line names are indicated to the left of each row. GM847 and JFCF-6/T.5K-sc1 cells were stained with antibodies recognising PML protein (red) and TRF2 protein (green), while U-2 OS cells were stained for PML protein (red) and telomeric DNA (green). 4′,6-diamidino-2-phenylindole (DAPI; blue) was used as a DNA counterstain. Scale bar indicates 10 μm.

Experimental variation in fluorescent labelling may lead to areas of an image with higher background than other regions. As a result, MatCol was developed to calculate individual background intensities of the red and green channels specifically within each ROI. This ROI- and channel-based processing allows robust and dynamic identification of different background luminance in various cells. Figure 2 shows nuclei from three cell lines that have different background. For example, the background fluorescence of the green channel in the JFCF-6/T.5K-sc1 nucleus is higher compared to the U-2 OS nucleus, making it difficult to discern signal from background in the JFCF-6/T.5K-sc1 nucleus. The entire image from which the JFCF-6/T.5K-sc1 nucleus was isolated has been included in order to demonstrate this is a rare nucleus with a high degree of background staining (Supplementary Figure S3). The nucleus can still be included in the analysis due to the method of background subtraction available in the MatCol program. Consequently, MatCol removed more background from the higher level of background in the green channel ROI so that all objects were clearly detected in the RGB image.

### Validation of colocalisations identified by MatCol

We selected three cell lines and immunofluorescently labelled the proteins PML and TRF2, or PML and telomeric DNA, as detailed in the Methods section. Colocalisations in a total of 12 images (five from GM847, four from JFCF-6/T.5K-sc1 and three from U-2 OS), which included 370 ROIs, were counted manually. Manual counting to quantitate colocalised objects is still considered the established “gold standard” even though the process is laborious, prone to human error and time-consuming. The results of manual counting were compared to those obtained by analysis using MatCol and CellProfiler^17^.

CellProfiler’s colocalisation pipeline (available from http://cellprofiler.org/examples/) was employed to compare MatCol’s ROI-based thresholding approach against the global thresholding methods available in the pipeline. The pipeline defines colocalisation based on touching or overlapping of two objects. If an object was touching multiple parent objects, the object was assigned to the parent with maximal overlap. Using the default setting of the pipeline seven different global thresholding methods were assessed: default (Otsu), Background, Kapur, MCT, MoG, Ridler Calvard and Robust background. The colocalisation results obtained using CellProfiler’s default thresholding method, Otsu, were significantly different from the manual counting (Wilcoxon matched pairs signed-rank two-tailed test; P = 0.0005; Supplementary Figure S4; Supplementary Table S1). The mean number of colocalisations identified by manual counting was 168 (median: 166), while the mean generated by the Otsu method was 1679 (median: 1563). Since there was a ten-fold difference between the Otsu method result and manual counts, we removed the Otsu results to avoid false significance values during multiple comparison post-hoc ranking^22^.

**Figure 3:**
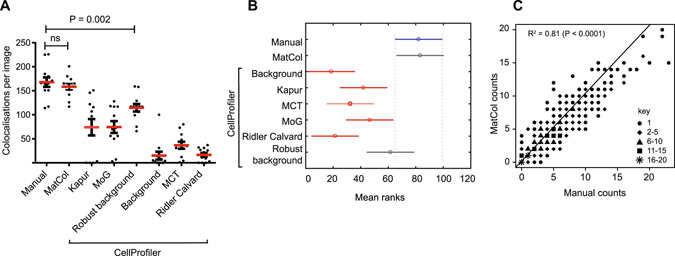
Comparison between the number of colocalisations by various methods. (**A**) Spread of total colocalisations in 12 images from three cell lines obtained by manual counting and automatically by either MatCol or CellProfiler using six different global thresholding methods: Kapur, MoG, Robust background, Background, MCT and Ridler Calvard. P = 0.002 comparing manual and Robust background CellProfiler results using Wilcoxon signed-rank test; ns = not significant when comparing manual and MatCol colocalisations using a Wilcoxon signed-rank test (P = 0.17). (**B**) Significance of difference in mean ranking by Kruskal Wallis followed by Tukey-Kramer post hoc test of the total number of colocalisations. C) Scatter plot of colocalisations detected in 370 ROIs total from three cell types identified by manual counting and MatCol. Note there are 111 unique values plotted on the graph, while 259 values overlapped as there were ROIs with the same manual and MatCol counts. Key defines the range of number of colocalisations. Linear regression analysis shows the line of best fit with R^2^ = 0.81 (P < 0.0001).

We next compared manual colocalisation results with the results generated by MatCol and CellProfiler using other six different global thresholding methods. There was a statistically significant difference between manual counting and the group consisting of MatCol and the CellProfiler results (Kruskal Wallis; P = 2.7 × 10^−12^). Further multiple comparison post-hoc testing using Tukey-Kramer revealed that results from five methods (Background, Kapur, MCT, MoG and Ridler-Calvard) were significantly different from the manual counting (Fig. 3A,B). The mean ranks of MatCol and Robust background were not significantly different as their comparison intervals overlap. Wilcoxon matched-pairs signed ranked two-tailed test confirmed that counting obtained from MatCol was not significantly different from the manual (P = 0.17). However, there was a significant difference between the medians of the Robust background method versus manual (Wilcoxon test; P = 0.002), demonstrating that use of the Robust background global thresholding method is not ideal. This analysis showed that, at an image level, MatCol results were similar to that identified manually.

We further studied the correlation between MatCol counting for each ROI (nucleus) against manual, since, in telomere biology it is important to know how many nuclei contain objects that are positively colocalised. The MatCol GUI reports the total number of colocalised objects in an image and in an ROI visually, but does not write output to a text file. Therefore, we used MatCol command-line utility (matcolcli.m) to run the tool on all images and the results for each ROI were transcribed to a text file. Plotting the number of colocalisations identified via manual counting versus MatCol (from all cell lines examined) shows a linear pattern, as described by the linear regression line of best fit with R^2^ = 0.81 (P < 0.0001; Fig. 3C). The highly significant P-value indicates that the correlation between MatCol and manual counting occurs more often than by random chance. Moreover, when control images were quantified using MatCol, and the results included in the data set used to generate Fig. 3C, the R^2^ value was not significantly changed (R^2^ = 0.83). These data suggest a strong agreement between manual and MatCol quantification with ROI-based thresholding - thus validating that MatCol could be used to accurately quantify signal colocalisations. In contrast, global thresholding, as commonly applied in many other software packages and CellProfiler, resulted in poor agreement between manual and MatCol quantification in our application to telomere biology.

### Modular software design

**Figure 4:**
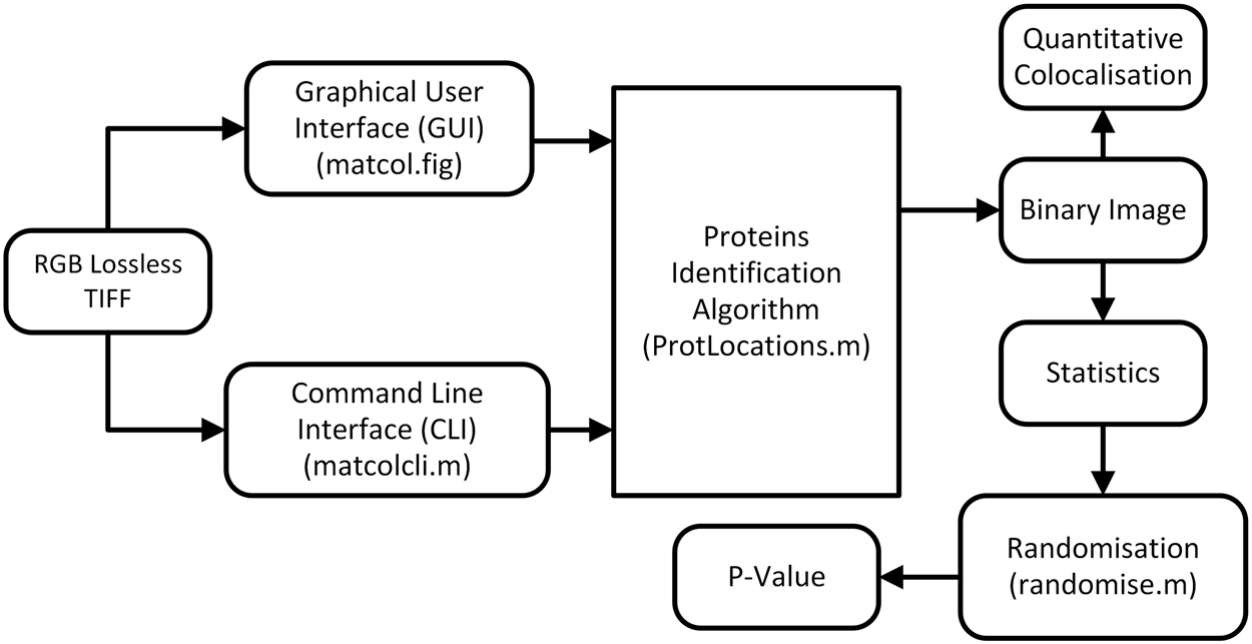
An overview of the MatCol software design in MATLAB. The main code file responsible for the execution of the identified component of the workflow is specified within brackets.

The modular design of MatCol makes it easy to reuse the code components (Fig. 4). The GUI is more appropriate for the analysis of a few images, while an alternative command-line interface can be used to automate the batch processing of a large number of images. In this case, results are written to a tab-delimited text file.

## Discussion

In recent years, computational algorithms have played a vital role in unveiling systems biology. Image analysis techniques have been widely used to identify the colocalisation of two fluorescently labelled targets, which is a necessary first step in determining whether the two targets interact with each other. Colocalisation identification has generally been performed manually with the assistance of image analysis software. Advances in microscopy make it easier to automatically generate a large number of images. In our study, requiring large cell numbers, images were taken using widefield microscopy. Rather than confocal Z-stack images, widefield microscopy of the thin cytocentrifuged samples was considered an appropriate tool to compare manual and MatCol quantification. Out of focus blurring is inherently low in thin cytospins. Moreover, compared to the photomultiplier tube (PMT) detectors in confocal microscopes, the larger dynamic range of the charge coupled device (CCD) detector used in widefield microscopes is more suitable for quantification. Thus, researchers require new methods to rapidly and accurately analyse colocalisations. In addition, manual colocalisation quantification can be subjective and prone to human error.

In many colocalisation studies, researchers illustrated ROIs by hand, presented colocalisations as scatter plots, and tried to deduce biology based on the value of various coefficients determined from these scatter plots^23, 24^. This method may be suitable for some cell biology studies, however, for biological studies where large numbers of objects require a precise count of colocalisation of two proteins, a more objective and automated method is required^16^. Furthermore, automatic global or manual thresholding to remove the background has been criticised and is controversial^25^. ImageJ and CellProfiler have many thresholding methods available. Use of the default thresholding methods in ImageJ or CellProfiler did not adequately remove background in our images, however, ImageJ and CellProfiler are still the preferred platforms for the calculation of pixel-intensity based coefficients. Since there was no default object-based colocalisation counting module in ImageJ, we compared MatCol counting against CellProfiler’s colocalisation pipeline. The CellProfiler pipeline offers a large range of thresholding methods, and as such, we did not test every possible configuration. Therefore, it is possible that a particular configuration of CellProfiler would provide a similar result to MatCol. MatCol uses local ROI-based background thresholding. If there are ROIs in an image with different levels of background, MatCol will remove more or less background from the ROI, according to the level of background luminance in the ROI. The thresholding method used by MatCol (Equation 1) required designing a multiplier factor. In all our test settings, a multiplier factor of two worked well. However, instead of hard coding this number into MatCol we made this available as a configurable setting for investigators. In comparison, manual thresholding, that many tools offer, applies a single cut-off across the image and needs to be adjusted for every image, whereas our multiplier factor was designed to work well for all images in an experiment. We acknowledge that results obtained by MatCol, as well as other tools, will vary if parameters in the tools are modified.

We analysed 12 images having 370 nuclei to validate the accuracy of MatCol across three different cell lines under varying conditions. Finding a universal algorithm that could remove background and identify signal locations in the three cell lines was challenging as some of the tested images had different levels of background. The green fluorescent channel background in the JFCF-6/T.5K-sc1 nuclei shown in Fig. 2 was high compared to other nuclei in the same field of view (Supplementary Figure S3), making separation of signal from background difficult. In such a case, manual counting of colocalisations would also be subjective. In fluorescence microscopy variation in signal to noise ratio is also acknowledged, and as a consequence MatCol misidentified some objects resulting in some disagreement in certain ROIs (Fig. 3C). Nevertheless, comparisons between MatCol and manual counting showed that the MatCol algorithm identified signal colocalisations in all three cell lines (Fig. 3) as evidenced by the high correlation observed between the manual and MatCol counting. This validation also showed that default MatCol values for median, Wiener and threshold multiplier performed well on a range of cells; therefore, we submit MatCol as a tool to count colocalised objects.

MatCol is a specialised tool and, unlike ImageJ or CellProfiler, it was designed to produce reproducible measurements and increase the speed at which object-based colocalisations can be quantified. MatCol reports colocalisations as the number of objects found to overlap between two channels. This number could be saved in a database which could allow machine learning of large scale analysis among different cell-lines and treatment conditions^26, 27^. The thresholding method used by MatCol (Equation 1) is simple to understand and implement in other tools, if the maintainability of MatCol becomes an issue in the future.

MatCol calculates the significance of colocalisations quantified within an image as a P value: an easily interpreted measure of significance. This option is only useful when analysing whether signal overlaps are indeed real or a consequence of random chance in a single image, however this is a rarely used feature in biological settings where generally large number of images are quantified. In order to compute the statistical difference between two groups (e.g. control versus treatment), MatCol colocalisation counts from a larger set of images should be analysed for statistical significance using other tools. Colocalised objects in our analysed images were small, generally 10–15 pixels. Therefore, randomising within a much larger ROI (>20,000 pixels) provided us a working spatial ratio of ≤1:667. The P value will not be a valid indicator of significance if the object to ROI ratio is greater than the object to ROI ratio used in our analysis. We recommend the ROI be >600-fold larger than the object of interest. We used a minimum of 3 pixels overlap in our validation example which is 20–30% overlap for our objects having general size of 10–15 pixels. We consider this a weak proximity assay for studying colocalisation of objects, however, a researcher can make the criteria stringent by requiring more pixels overlap for larger objects. In conclusion, MatCol is a novel, open source and user-friendly tool that addresses the needs of studying the object-based counting of colocalisations of two biological features.

## Methods

### Cell culture

JFCF-6/T.5K-sc1 and GM847 cells were described previously^28, 29^. U-2 OS cells were obtained from American Type Culture Collection. All cultures were maintained in Dulbecco’s modified Eagle media supplemented with 10% foetal bovine serum in a 37 °C, 10% CO_2_ incubator.

### Immunofluorescence staining and imaging

Single cell suspensions of 7 × 10^4^ cells were cytospun onto SuperFrost Plus microscope slides using a Shandon Cytospin 4. The thin cytocentrifuged samples with nuclei flattened to a similar depth as the cytoplasm (approximately 3 µm) were optimal for imaging large numbers of nuclei using widefield microscopy. Fixation was performed using 4% formaldehyde in PBS and permeabilisation with 0.1% Triton X-100 in PBS. Cells were then simultaneously blocked and RNase A-treated by incubation with 0.1 mg/mL RNase A diluted in antibody dilution buffer (ABDIL; 20 mM Tris-HCl, pH 7.5, 0.2% fish gelatin, 2% BSA, 0.1% Triton X-100, 150 mM NaCl, 0.1% sodium azide). JFCF-6/T.5K-sc1 and GM847 cells were stained with antibodies specific for TRF2 and PML diluted in ABDIL for 2 hr at 37 °C in a humidified chamber. Subsequent to washes in PBS with 0.1% Tween-20 (PBST), cells were stained with appropriate Alexa Fluor secondary antibodies (donkey anti-goat 647 [PML] and donkey anti-mouse 594 [TRF2]) for 30 min at 37 °C in a humidified chamber. After an additional set of PBST washes, DNA was counterstained with DAPI, and cells were mounted using DABCO anti-fade mounting media^29^. For U-2 OS cells, cells were stained with an antibody against PML and the secondary antibody donkey anti-goat 647, as detailed above. Fixation was then performed with 4% formaldehyde in PBS in order to fix the antibody staining. Cells were rinsed twice in milliQ H_2_O (mqH_2_O), dehydrated in an ethanol series (2 min each in 70, 90 and 100% ethanol) and allowed to air dry. The cells were then overlaid with FITC-conjugated telomere probe, denatured at 80 °C for 3 min and incubated in the dark at room temperature (RT) for 3 hr. Following washes in 65 °C and RT 2x SSC/0.1% Tween 20, the cells were counterstained with DAPI and mounted in DABCO^30^. DAPI was used to identify individual nuclei (ROIs). Control slides were prepared in a similar manner, apart from exclusion of primary antibodies i.e. cells were cytospun, fixed, permeabilised, incubated with secondary antibodies, and DAPI counterstained.

Acquisition of digital images was performed using AxioImager.Z2 (pixel size 0.144 µm × 0.144 µm) or Axio Imager M1 (pixel size 0.102 µm × 0.102 µm) widefield (epifluorescence) microscopes with AxioCam MRm monochrome CCD cameras and HXP-120C mercury short-arc fluorescence illuminator lamps (Zeiss, Germany). Images were acquired and processed using ZEN 2 software (Zeiss) and saved as RGB (red, green, blue) lossless TIFF files. Filter sets specific for each fluorescent image included DAPI (excitation [ex] 365, emission [em] BP 445/50), FITC (ex 475/40, em BP 530/50), Alexa Fluor 594 (ex 560/40, em BP 630/75), and Alexa Fluor 647 (ex 640/30, em BP 690/50). Plan-Apochromatic 63X oil immersion objectives (NA 1.4) were used.

### MatCol algorithm

MatCol is written in MATLAB 2016b (Mathworks, USA) and can be obtained from http://bioinformatics.cmri.org.au with test images. MatCol identified signal locations within each ROI where luminance exceeds local threshold value *T*, where *T* is calculated as:

1 $$T=[{\rm{\mu }}]+M\,[{\rm{\sigma }}]$$

In the above equation, [μ] is the rounded mean to the least integer greater than or equal to the mean (ceiling rounding), M is a user-defined multiplier factor and [σ] is the ceiling rounded standard deviation of pixel intensities in an ROI. For example, if the mean intensity (μ) within an ROI is 25 and standard deviation (σ) is 3, then for M = 2 (the MatCol default value), all pixels having a value less than 31 are considered as background. We have used the blue channel to mark ROIs by the Otsu thresholding method^31^. We used ‘8-connected’ to define pixel connectivity in segmenting connected objects. This means that pixels with coordinates (x ± 1, y ± 1) are considered connected to a pixel with coordinate (x,y).

The lateral resolution is the smallest distance two spots can be resolved in a two-dimensional image. This minimum resolvable distance is calculated using the Rayleigh Criterion (d) = 0.61λ/NA (λ is the wavelength of emitted light, and NA is the numerical aperture of the objective). For our analysis using the longer emission wavelength, d = 0.29 µm and with a pixel size of 0.14 µm, the minimum spatial overlap of two objects requires three pixels. For validation analysis presented in the Results section, a colocalisation is defined as at least three pixels in each red and green channel occupying the same space. In effect, colocalisation in which three overlapping pixels is our minimum requirement is effectively a weak proximity assay. The number of pixels defining a colocalisation can be modified by the user in the Configuration tab (Supplementary Figure S2). When two objects in a channel overlap with an object in another channel, two colocalisations are reported. Object size restrictions in pixels can also be defined in the Configuration tab.

We provide the widely used median and Wiener filters in the GUI allowing the user to fine-tune the removal of background^32^. Both of these filters require specifying a window, and based on our testing we recommend the default window sizes of 3 × 3 for the median filter and 5 × 5 for the Wiener filter. The non-linear median filter selects the middle value within a specified window and thus removes any outlier intensities. This filter is widely used to remove “salt and pepper” type noise within an image. In contrast, the Wiener filter is an adaptive linear filter that applies less smoothing to an image area where variance in a window is high and applies greater smoothing where variance is low^33^. The mean (μ) and variance (σ^2^) value of pixels *a* within a window of size N × M is calculated by the following formulas^34, 35^:

2 $${\rm{\mu }}=\,\frac{1}{NM}\sum _{{n}_{1},{n}_{2}\in \eta }a({n}_{1},{n}_{2})\,$$

3 $${{\rm{\sigma }}}^{2}=\,\frac{1}{NM}\sum _{{n}_{1},{n}_{2}\in \eta }{a}^{2}({n}_{1},{n}_{2})-{\mu }^{2}\,$$

where *n*_1_ and *n*_2_ are coordinates of pixel set *η*. Noise variance *v* ^2^ is calculated by the average of all local calculated variances and the Wiener value for each pixel ‘b’ is calculated by:

4 $${\rm{b}}({n}_{1},\,{n}_{2})=\mu +\,\frac{{{\rm{\sigma }}}^{2}-{v}^{2}}{{{\rm{\sigma }}}^{2}}({\rm{a}}({n}_{1},\,{n}_{2})-\mu )$$

The ROI is identified using the blue channel. In order to use other channels as the ROI, the user needs to change channels before feeding the images through MatCol. Other third party software such as ImageJ can be used to change channel colours.

### Statistics

The statistical significance of the MatCol computed colocalisation is calculated by Monte Carlo simulation^36^, which randomises the detected red and green channel objects within all ROIs in their respective channels. During randomisation, the shape and size of the segmented objects are kept the same however they are allowed to overlap as in a true randomisation process when two objects can overlap, resulting in a larger object. Objects are not separated if they appear to overlap, as the tool is best configured to study small objects as described in our biological application. The colocalisation of this randomness is calculated and the process is repeated to acquire a user-specified number of random results. These randomly calculated colocalisation numbers were compared against the actual measured colocalisation by the Student’s t-test to generate a P-value. This P-value estimates the statistical significance of the experimental observed colocalisation against random chance of obtaining the same colocalisation count, and is performed on a single image.

Statistical tests for the validation study described in the results section were performed using GraphPad Prism 5.04 and MATLAB. Nonparametric statistical tests (Kruskal Wallis and Wilcoxon) were chosen because the data were not normally distributed.

## Electronic supplementary material

Supplementary Material
