# Supplementary material for: MatCol: a tool to measure fluorescence signal colocalisation in biological systems
Source: Sci Rep. 2017 Aug 21;7:8879. doi: 10.1038/s41598-017-08786-1 (PMC5566543; doi:10.1038/s41598-017-08786-1)
Supplement: Supplementary file 1 — Supplementary Material [file 41598_2017_8786_MOESM1_ESM.doc]

# MatCol: a tool to measure fluorescence signal colocalisation in biological systems

Matloob Khushi^1^*, Christine E. Napier^2^, Christine M. Smyth^3^, Roger R. Reddel^2^ and Jonathan W. Arthur^1^

^1^Bioinformatics Unit, Children’s Medical Research Institute, The University of Sydney, Westmead, NSW, Australia.

^2^Cancer Research Unit, Children’s Medical Research Institute, The University of Sydney, Westmead, NSW, Australia.

^3^Gene Therapy Unit, Children’s Medical Research Institute, The University of Sydney, Westmead, NSW, Australia.

*Correspondence to mkhushi@uni.sydney.edu.au.

**Supplementary Material**


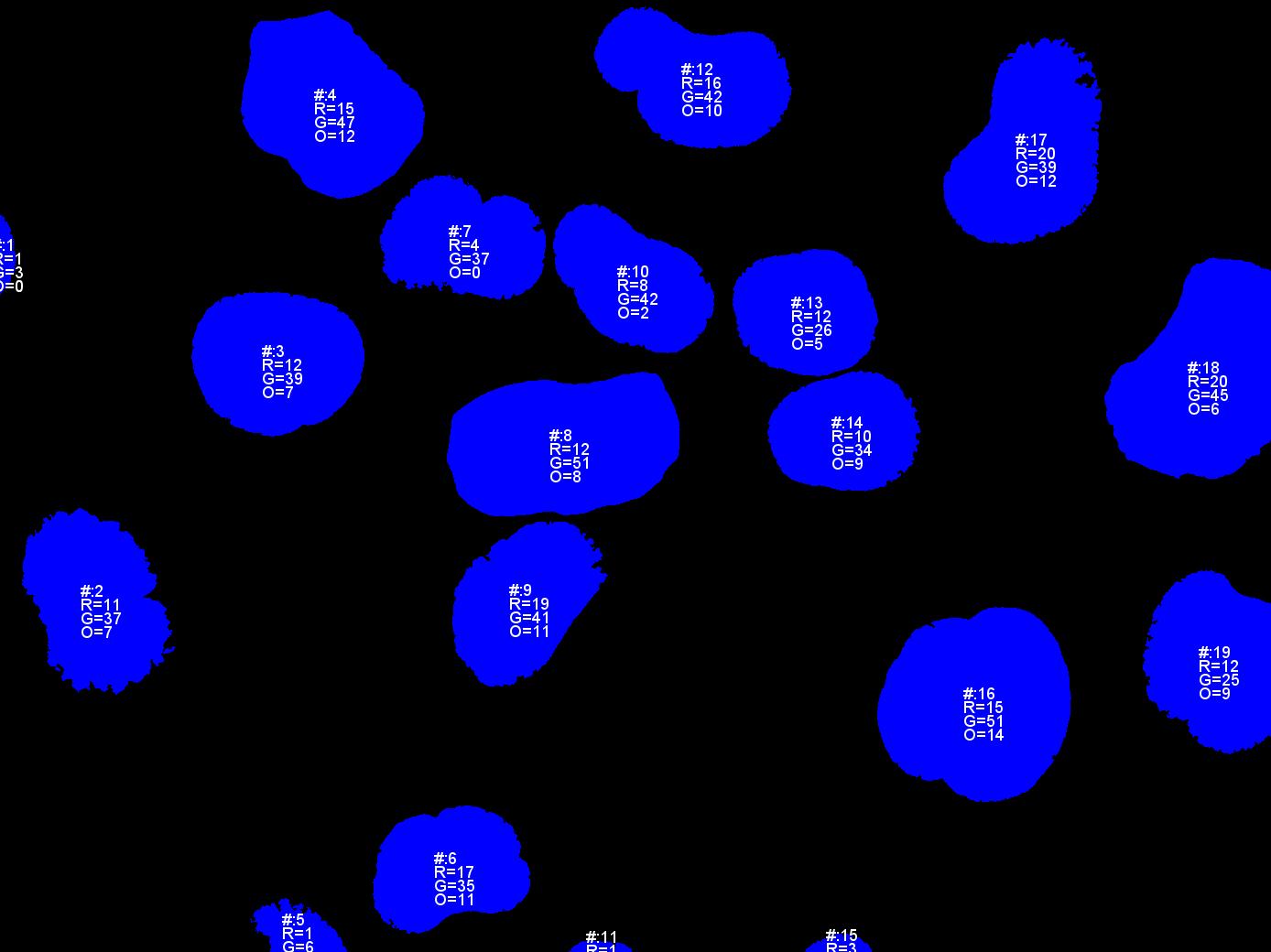


**Supplementary Figure S1. Entire image from which zoomed region of Figure 1 was obtained.** To obtain the image presented in Figure 1, the above image was analysed, and then zoomed into a single ROI (#3) for ease of viewing. The masks enumerate the ROI (#), red (“R”) and green (“G”) signals, as well as their overlap (“O”) in each ROI.


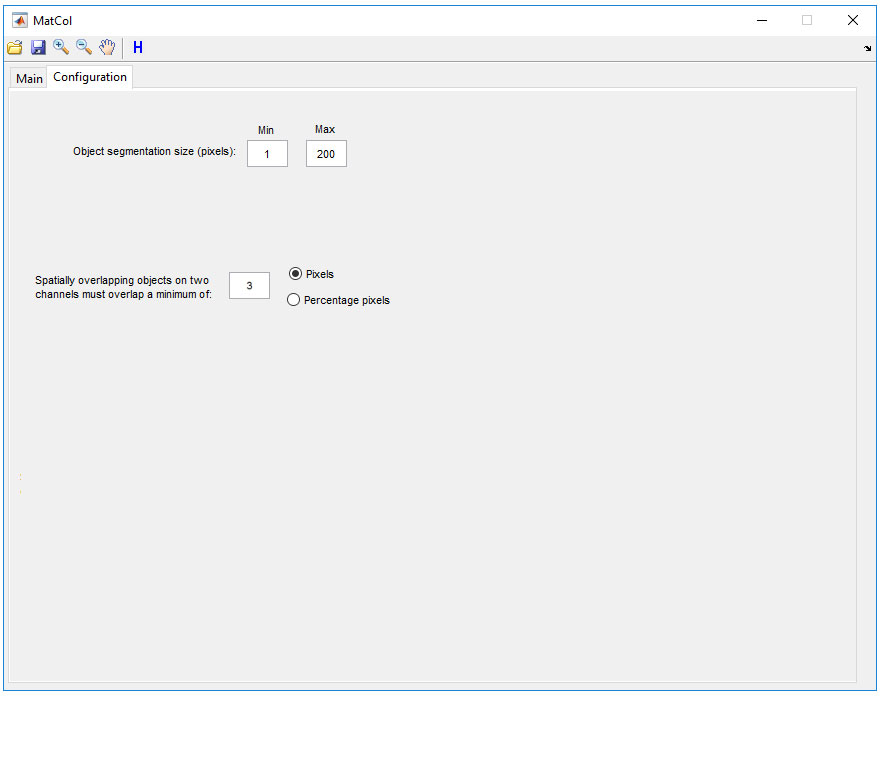


**Supplementary Figure S2. Configuration tab of MatCol.** MatCol can be configured to restrict object segmentation to within a certain pixel size. The minimum number of overlapping pixels, or the percentage of total pixels, required to define a colocalisation between two channels can also be set by the user. For our analyses, colocalised objects were identified when there was minimum of three overlapping pixels.

The default MatCol settings identify ROIs from the blue channel. If ROIs need to be selected from either red or green channel, then the channels can be changed using other tools. This can be achieved either using microscope software such as Zen (Zeiss) or free tools such as Fiji/ImageJ. For example, in Fiji, channels can be changed within an RGB image by separating channels using the “Split Channels” option from the Image 🡪 Color menu. Three channels become three separate grey scale images, and these images can be merged as channels of a new RGB image using the “Merge Channels” option.

**Supplementary Information S1**: Change of a channel for the ROI selection.


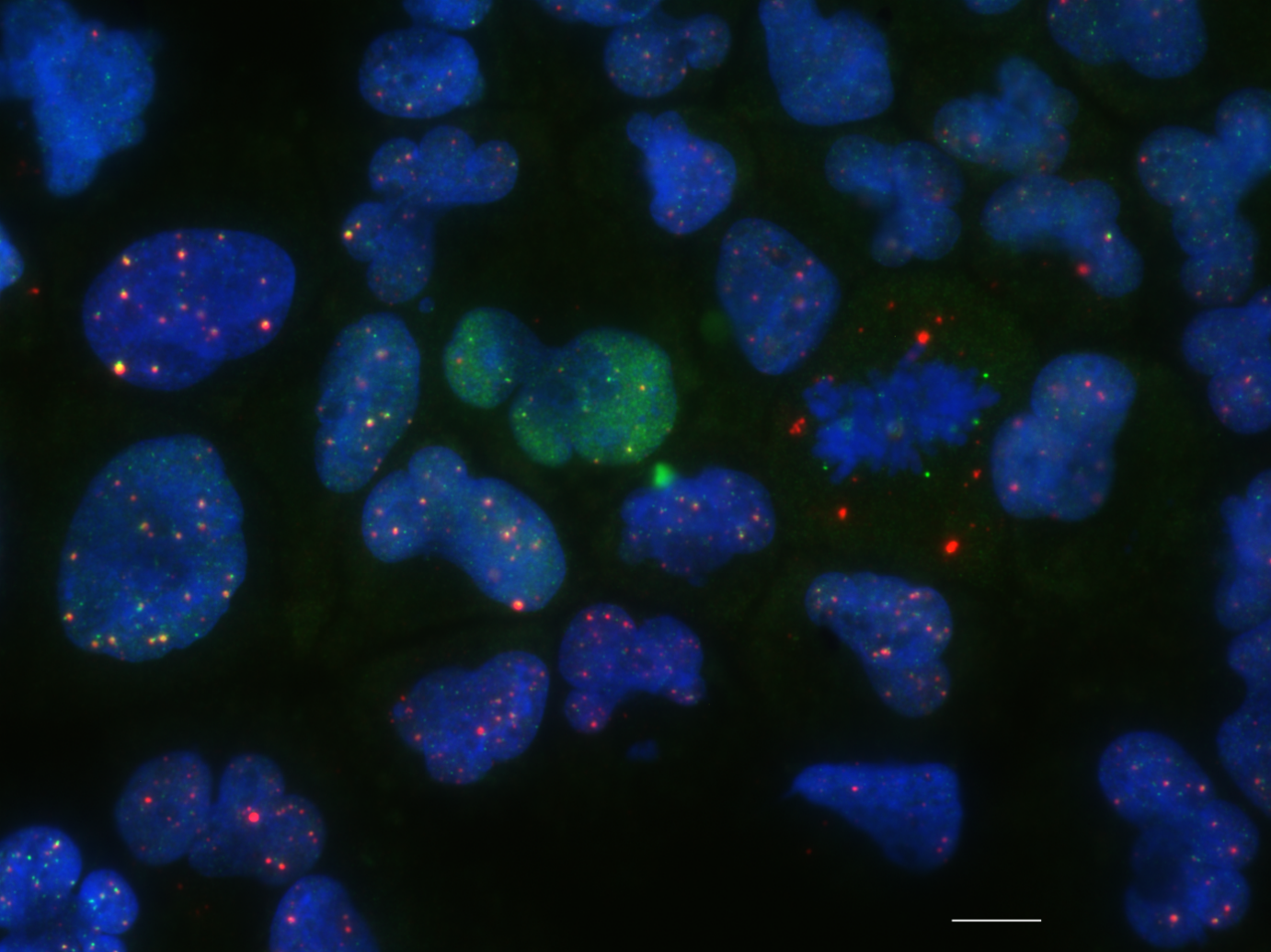


**Supplementary Figure S3. Entire image from which JFCF-6/T.5K-sc1 nuclei in Figure 2 was obtained.** The background fluorescence of the green channel in a single JFCF-6/T.5K-sc1 nucleus is greater compared to the remaining nuclei. PML protein was stained in red, TRF2 protein in green, and DAPI in blue. Scale bar indicates 10 μM.


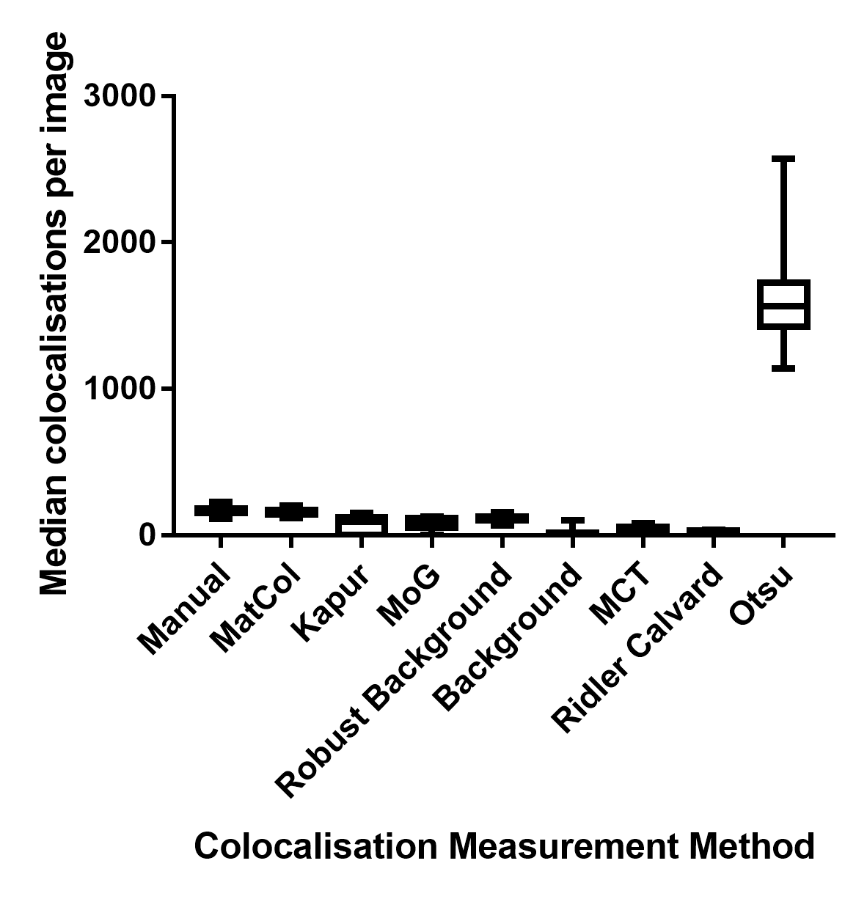


**Supplementary Figure S4. Box plots of the median number of colocalisations quantified by method indicated on X-axis.** The median number of colocalisations in 12 images from three cell lines obtained by manual counting, MatCol, or CellProfiler using seven different global thresholding methods: Kapur, MoG, Robust background, Background, MCT, Ridler Calvard, and Otsu. The box body represents interquartile range of the colocalisation counts while the whiskers indicate maximum and minimum values.

|  |  | **Colocalisation counting method** | | | | | | | | |
| --- | --- | --- | --- | --- | --- | --- | --- | --- | --- | --- |
|  |  | **Manual** | **MatCol** | **CellProfiler global thresholding method** | | | | | | |
|  |  |  |  | **Background** | **Kapur** | **MCT** | **MoG** | **Ridler Calvard** | **Robust background** | **Otsu** |
| **Sample image** | GM847-1 | 117 | 117 | 0 | 59 | 48 | 56 | 9 | 117 | 1590 |
|  | GM847-2 | 151 | 126 | 16 | 122 | 12 | 73 | 11 | 117 | 1535 |
|  | GM847-3 | 185 | 158 | 5 | 151 | 31 | 50 | 22 | 117 | 1501 |
|  | GM847-4 | 165 | 152 | 8 | 107 | 29 | 56 | 27 | 129 | 1403 |
|  | GM847-5 | 180 | 161 | 2 | 97 | 4 | 7 | 3 | 158 | 2571 |
|  | JFCF-6/T.5K-1 | 171 | 142 | 0 | 0 | 1 | 3 | 31 | 159 | 1730 |
|  | JFCF-6/T.5K-2 | 164 | 159 | 14 | 3 | 30 | 117 | 32 | 117 | 1287 |
|  | JFCF-6/T.5K-3 | 166 | 174 | 0 | 87 | 80 | 118 | 37 | 99 | 1710 |
|  | JFCF-6/T.5K-4 | 114 | 165 | 31 | 116 | 59 | 128 | 26 | 116 | 1492 |
|  | U-2 OS-1 | 226 | 180 | 4 | 0 | 37 | 70 | 0 | 66 | 2569 |
|  | U-2 OS-2 | 151 | 165 | 100 | 146 | 35 | 117 | 0 | 75 | 1140 |
|  | U-2 OS-3 | 225 | 201 | 0 | 0 | 73 | 98 | 0 | 106 | 1623 |
|  | **Mean** | 168 | 158 | 15 | 74 | 37 | 74 | 17 | 115 | 1679 |
|  | **Median** | 166 | 160 | 5 | 92 | 33 | 72 | 17 | 117 | 1563 |

**Supplementary Table S1:** Colocalisation count using the indicated methods. Use of the default global thresholding method (Otsu) with CellProfiler gave the highest colocalisation count.
